# Virulence Gene Sequencing Highlights Similarities and Differences in Sequences in *Listeria monocytogenes* Serotype 1/2a and 4b Strains of Clinical and Food Origin From 3 Different Geographic Locations

**DOI:** 10.3389/fmicb.2018.01103

**Published:** 2018-06-05

**Authors:** Sofia V. Poimenidou, Marion Dalmasso, Konstantinos Papadimitriou, Edward M. Fox, Panagiotis N. Skandamis, Kieran Jordan

**Affiliations:** ^1^Laboratory of Food Quality Control and Hygiene, Department of Food Science and Human Nutrition, Agricultural University of Athens, Athens, Greece; ^2^Teagasc Food Research Centre, Moorepark, Fermoy, Co., Cork, Ireland; ^3^Laboratory of Dairy Research, Department of Food Science and Human Nutrition, Agricultural University of Athens, Athens, Greece; ^4^CSIRO Agriculture and Food, Werribee, VIC, Australia

**Keywords:** *Listeria monocytogenes*, virulence, gene sequencing, diversity, *prfA*, *hly*, *actA*

## Abstract

The *prfA*-virulence gene cluster (*p*VGC) is the main pathogenicity island in *Listeria monocytogenes*, comprising the *prfA, plcA, hly, mpl, actA*, and *plcB* genes. In this study, the *p*VGC of 36 *L. monocytogenes* isolates with respect to different serotypes (1/2a or 4b), geographical origin (Australia, Greece or Ireland) and isolation source (food-associated or clinical) was characterized. The most conserved genes were *prfA* and *hly*, with the lowest nucleotide diversity (π) among all genes (*P* < 0.05), and the lowest number of alleles, substitutions and non-synonymous substitutions for *prfA*. Conversely, the most diverse gene was *actA*, which presented the highest number of alleles (*n* = 20) and showed the highest nucleotide diversity. Grouping by serotype had a significantly lower π value (*P* < 0.0001) compared to isolation source or geographical origin, suggesting a distinct and well-defined unit compared to other groupings. Among all tested genes, only *hly* and *mpl* were those with lower nucleotide diversity in 1/2a serotype than 4b serotype, reflecting a high within-1/2a serotype divergence compared to 4b serotype. Geographical divergence was noted with respect to the *hly* gene, where serotype 4b Irish strains were distinct from Greek and Australian strains. Australian strains showed less diversity in *plcB* and *mpl* relative to Irish or Greek strains. Notable differences regarding sequence mutations were identified between food-associated and clinical isolates in *prfA, actA*, and *plcB* sequences. Overall, these results indicate that virulence genes follow different evolutionary pathways, which are affected by a strain's origin and serotype and may influence virulence and/or epidemiological dominance of certain subgroups.

## Introduction

*Listeria monocytogenes* is a facultative intracellular foodborne pathogen, with pregnant women and neonates, immunocompromised individuals, and the elderly representing high risk groups for infection (Farber and Peterkin, 1991; EFSA ECDC, 2015). It is equally capable of both a saprophytic lifecycle in the environment and human infection causing the severe disease of listeriosis (Gray et al., 2006). Due to its wide variety of reservoirs (Farber and Peterkin, 1991; Lianou and Sofos, 2007), its ability to colonize abiotic surfaces (Møretrø and Langsrud, 2004; Poimenidou et al., 2016b) and to withstand environmental stresses (Hill et al., 2002; Poimenidou et al., 2016a), it is frequently implicated in food processing plant contamination, where it is able to persist for several months or years (Halberg Larsen et al., 2014), thus raising the risk to food safety. After transmission via contaminated food to humans, *L. monocytogenes* cells may cause illnesses such as gastroenteritis or invasive listeriosis following intestinal translocation. It may then be carried by blood or lymph fluid and reach the mesenteric lymph nodes, spleen and/or the liver, leading to subclinical pyogranulomatous hepatitis, meningoencephalitis, septicemia, placentitis, abortion, or neonatal septicemia (Vázquez-Boland et al., 2001b). Within the host, *L. monocytogenes* parasitizes macrophages and invades non-phagocytic cells, utilizing its virulence factors to mediate cell-to-cell spread (de las Heras et al., 2011).

The virulence potential of *L. monocytogenes* relies on several molecular determinants (Camejo et al., 2011), which play key roles at different stages of the infection process. Among the early stages of the infection process, genes including the internalins (*inlA, inlB, inlF, inlJ*) play key roles in adhesion and invasion. Intracellular pathogenesis heavily relies on factors transcribed by genes located in the major *prfA*-regulated virulence gene cluster (*p*VGC), also referred to as *Listeria* pathogenicity island 1 or LIPI-1 (Vázquez-Boland et al., 2001a; Ward et al., 2004). *p*VGC genes facilitate the intracellular growth and spread of the bacterium in the host and consist of a monocistron *hly*, which occupies the central position in the locus, a lecithinase operon comprising *mpl, actA*, and *plcB* genes, which is located downstream from *hly* and transcribed in the same orientation, and the *plcA-prfA* operon located upstream from *hly* and transcribed in the reverse direction (Portnoy et al., 1992; Vázquez-Boland et al., 2001b; Roberts and Wiedmann, 2003). The *prfA* gene encodes the PrfA protein, which is required for the transcription of *p*VGC, and *prfA* itself. Listeriolysin O (LLO) encoded by the *hly* gene is a pore-forming toxin that mediates lysis of bacterium-containing phagocytic vacuole, resulting in the release of bacterial cells into the host cytoplasm. *plcA* and *plcB* encode the phosphatidylinositol-specific phospholipase C (PI-PLC) and zinc-dependent broad-spectrum phospholipase C (PC-PLC), respectively, which synergistically with LLO mediate the escape of the pathogen from the single- and double-membrane-bound vacuoles. After lysis, the intracellular motility and cell-to-cell spread are mediated by the surface protein actin A (ActA) through actin polymerization, for which additional functions (i.e., role in invasion, aggregation, colonization and persistence in the gut lumen) have been reported (Suárez et al., 2001; Travier et al., 2013). *mpl* encodes a zinc metalloproteinase needed to activate PC-PLC in order to initiate a new infection cycle.

*Listeria monocytogenes* is a genetically diverse species; its isolates form a structured population and are differentiated into four distinct lineages and 13 serotypes (Orsi et al., 2011), with the majority of isolates clustering into lineage I (serotypes 1/2b, 3b, 3c, 4b) and lineage II (serotypes 1/2a, 1/2c, 3a). Serotypes 4b and 1/2a are overrepresented among isolates associated with human listeriosis cases and food environment isolates, respectively (McLauchlin, 1990, 2004; Schuchat et al., 1991; Norton et al., 2001; Jacquet et al., 2002; Mereghetti et al., 2002; Gray et al., 2004; Lukinmaa et al., 2004; Gilbreth et al., 2005; Kiss et al., 2006; Swaminathan and Gerner-Smidt, 2007; Ebner et al., 2015). Additionally, various *L. monocytogenes* strains have presented diversity in virulence potential (Brosch et al., 1993; Chakraborty et al., 1994; Jaradat and Bhunia, 2003; Roche et al., 2003; Neves et al., 2008). Defective forms of virulence determinants were identified as the source of such virulence attenuation (Olier et al., 2002, 2003; Roberts et al., 2005; Roche et al., 2005; Témoin et al., 2008; Van Stelten et al., 2011).

The reasons that 1/2a serotype strains predominate among food environment isolates and 4b serotype strains among human listeriosis isolates are under investigation, with no clear inference made so far (Jaradat et al., 2002; Larsen et al., 2002; Gray et al., 2004; Jensen et al., 2007, 2008; Neves et al., 2008; Houhoula et al., 2012). On the other hand, there are indications of selective pressure for maintenance or specific adaptation of the *p*VGC genes in particular environments (Roberts et al., 2005; Orsi et al., 2008; Travier et al., 2013). Comparative genotyping could contribute to identifying unique genetic determinants toward the intraspecific pathogenic characteristics of *L. monocytogenes* isolates. Considering the above, the objective of this study was to examine the nucleotide diversity of the *p*VGC genes of *L. monocytogenes* strains isolated from human clinical cases and food or food-related environments, which belonged to the serotypes 4b and 1/2a and originated from three distinct geographical locations (i.e., Australia, Greece, and Ireland). Studying these variations may provide valuable information toward understanding the significance of virulence gene variation and the influence of environmental pressures acting on the genes.

## Materials and methods

### Bacterial strains

A total of 36 *Listeria monocytogenes* strains (Table 1) were analyzed in this study. The strains represented three distinct geographically dispersed regions (Australia, Greece, Ireland), two serotypes (serotype 4b and 1/2a) and two isolation sources (clinical and food–related isolates). The clinical strains were kindly provided by Dr. Josheph Papaparaskevas (Houhoula et al., 2012) and Prof. Martin Cormican (University College Hospital, Galway, Ireland). The food-associated isolates were obtained from food and the food-processing environment. The strains were serotyped using a combination of antisera specific to the *L. monocytogenes* somatic O-antigen (Denka Seiken Co., Ltd., Tokyo, Japan), in tandem with a PCR-based serovar determination assay (Doumith et al., 2004), as described by Fox et al. (2009). Bacterial strains were stored at −80°C in Tryptic Soy broth (TSB) containing 20% glycerol and were cultured in TSB supplemented with 0.6% yeast extract (YE) at 37°C overnight, prior to pulsed-field gel electrophoresis (PFGE) and DNA extraction.

**Table 1:** Origins and characteristics of 36 *L. monocytogenes* strains used in the study.

**Country**	**Isolate**	**Origin**	**Date**	**Serotype**
Ireland	IR_227	Dairy processing environment	2008	1/2a
Ireland	IR_728	Dairy processing environment	2012	1/2a
Ireland	IR_872	Dairy processing environment	2013	1/2a
Ireland	IR_873	Clinical isolate	2013	1/2a
Ireland	IR_874	Clinical isolate	2013	1/2a
Ireland	IR_875	Clinical isolate	2013	1/2a
Ireland	IR_250	Dairy processing environment	2008	4b
Ireland	IR_338	Dairy processing environment	2008	4b
Ireland	IR_355	Dairy processing environment	2008	4b
Ireland	IR_876	Clinical isolate	2013	4b
Ireland	IR_877	Clinical isolate	2013	4b
Ireland	IR_878	Clinical isolate	2013	4b
Greece	GR_PL11	Chicken	2007	1/2a
Greece	GR_PL18	Chicken	2007	1/2a
Greece	GR_PL37	Clinical isolate	2009	1/2a
Greece	GR_PL38	Clinical isolate	2009	1/2a
Greece	GR_PL44	Clinical isolate	2013	1/2a
Greece	GR_PL50	Food isolate	2013	1/2a
Greece	GR_PL4	Dairy farm environment	2007	4b
Greece	GR_PL13	Chicken	2007	4b
Greece	GR_PL32	Clinical isolate	2009	4b
Greece	GR_PL41	Clinical isolate	2009	4b
Greece	GR_PL46	Clinical isolate	2013	4b
Greece	GR_FL78	Meat	2012	4b
Australia	AU_2884	Seafood	2009	1/2a
Australia	AU_2919	Meat processing environment	2013	1/2a
Australia	AU_2942	Dairy food	2009	1/2a
Australia	AU_2994	Vegetable	2011	1/2a
Australia	AU_2998	Meat	2011	1/2a
Australia	AU_Lm14-002	Dairy food	2014	1/2a
Australia	AU_2473	Dairy food	1998	4b
Australia	AU_2544	Clinical isolate	1994	4b
Australia	AU_2727	Meat	1988	4b
Australia	AU_2948	Dairy food	2010	4b
Australia	AU_2993	Dairy processing environment	2009	4b
Australia	AU_2995	Dairy processing environment	2009	4b

### PFGE of *L. monocytogenes* isolates

PFGE was carried out using the International Standard PulseNet protocol (Pulsenet USA, 2009). Two restriction enzymes, *Asc*I and *Apa*I, were used and band patterns were analyzed using Bionumerics version 5.10 software (Applied Maths, Belgium), as previously described (Fox et al., 2012). Briefly, band matching was performed using the DICE coefficient, with both optimization and tolerance settings of 1%. Dendrograms were created using the Unweighted Pair Group Method with Arithmetic Mean (UPGMA). Strains were considered to be indistinguishable when their pulsotypes displayed 100% similarity on the dendrogram and after confirmation by visual examination of the bands. To help support population diversity, all isolates were confirmed as having a unique pulsotype relative to any other isolate included in this study.

### DNA extraction

Following overnight culture of each strain, DNA was extracted using a DNeasy Blood and Tissue kit (Qiagen, UK) for strains from both Greece and Australia, or the QIAmp DNA mini kits (Qiagen) for strains from Ireland. A cell lysis step preceded DNA extraction and consisted in incubation of the cells in lysis buffer (20 mM TrisHCl, pH 8; 2 mM EDTA, pH 8; 1.2% Triton® −100; 20 mg/ml lysozyme) for 1 h at 37°C. DNA was stored at −20°C before use.

### Nucleotide sequencing of *actA, hly, mpl, plcA, plcB, prfA*

PCR amplification of the targeted genes was performed using genomic DNA extracted as described above. Primer design was based on available sequences of the targeted genes in public databases using Primer3Plus software version 2.3.5 (Untergasser et al., 2012). The primers and PCR conditions, all including 35 cycles, are described in Table 2. Phusion® High-Fidelity DNA polymerase (New England Biolabs® Inc, USA) and AccuTaq^TM^ LA DNA polymerase (Sigma, USA) were used for PCR reactions on 50 ng DNA for strains from Greece and Ireland, respectively. Following amplification, PCR products were purified using MinElute Gel Extraction kit (Qiagen). DNA sequencing was performed using external forward and reverse PCR primers at CEMIA SA (Larisa, Greece) and Source Biosciences (Dublin, Ireland) for Greece and Ireland PCR products, respectively. In the case of Australian isolates, sequences were extracted *in silico* from draft genomes using the same primer sets (Table 2) with Geneious® software version 9 (Kearse et al., 2012). DNA sequencing chromatograms were saved as ABI files for analysis.

**Table 2:** Primer sequences and PCR conditions for each virulence gene target.

**Gene**	**Strains ID**	**Primer sequence (5′-3′)**	**Hybridization temperature (°C)**	**Elongation time (min)**
*actA*	All strains	Forward^a^, GTATTAGCGTATCACGAGGA	60	1
		Reverse^a^, CAAGCACATACCTAGAACCA		
		Forward^b^, AAGMGTCAGTTRYGGATRCT	57	1
		Reverse^b^, CCCGCATTTCTTGAGTGTTT		
*hly*	227, 728, 872, 873, 874, 875, 876	Forward^a^, GGCCCCCTCCTTTGATTAGT	60	2
		Reverse^a^, GCCTCTTTCTACATTCTTCACAAA		
	355	Forward^a^, TATGCTTTTCCGCCTAATGG	57	1
		Reverse^a^, CGTGTGTGTTAAGCGGTTT		
	250, 338, 877, 878	Forward^a^, AAAAGAGAGGGGTGGCAAAC	60	2
		Reverse^a^, GCCTCTTTCTACATTCTTCACAAA		
	All strains	Forward^b^, CCAGGTGCTCTCGTRAAAGC	57	1
		Reverse^b^, RCCGTCGATGATTTGAACTT		
*mpl*	All strains	Forward^a^, GCCACCTATAGTTTCTACTGCAAA	57	1
		Reverse^a^, TGRAGAATTAAKTTTTCTCTAACATTT		
	All strains	Forward^b^, ATACGCTCGCGCTAAGTTCT	60	1
		Reverse^b^, GCTTCTTATTCGCCCATCTCG		
*plcA*	All strains	Forward^a^, ATCAAAGGAGGGGGCCATT	60	1
		Reverse^a^, CCGAGGTTGCTCGGAGATATAC		
*plcB*	All strains	Forward^a^, ATTGGCGTGTTCTCTTTAGG^c^	57	1
		Reverse^a^, CAAAGAAAAAGATTAACCTCCCTTT		
*prfA*	All strains	Forward^a^, TTCAGGTCCKGCTATGAAAC	57	1
		Reverse^a^, AACTCCATCGCTCTTCCAGA		

a *External primers located in upstream and downstream regions surrounding the targeted gene*.

b *Internal primers*.

c *Roche et al., 2005*.

### Data analysis

Sequence assembly was performed using SeqMan Pro application in Lasergene® Genomics suite (DNASTAR, USA). Geneious® software version 9 (Kearse et al., 2012) was used to construct translation alignments for each gene separately and the *p*VGC (a concatenated sequence comprising the *prfA, plcA, hly, mpl, actA, plcB* sequences).

### Descriptive analysis

Number of polymorphic sites (S), nucleotide diversity (π; average pairwise nucleotide differences per site), number of segregating sites (θ), and Tajima's *D* for neutrality were calculated using DnaSP software version 5 (Librado and Rozas, 2009). Number of polymorphic sites, number of substitutions, number of synonymous substitutions (SS) and non-synonymous substitutions (NSS), and the G + C content (%) were defined using Geneious software version 9 (Kearse et al., 2012). The d_N_/d_S_ ratios or ω [number of non-synonymous substitutions/nonsynonymous sites (d_N_) to the number of synonymous substitutions/synonymous sites (d_S_)] were calculated using the Datamonkey online platform (Kosakovsky Pond and Frost, 2005). 3D scatterplots were created using 'Excel 3D Scatter Plot' version 2.1 (available at: http://www.doka.ch/Excel3Dscatterplot.htm).

### Phylogenetic analysis

Phylogenetic trees were generated using the NeighborNet algorithm (Bryant and Moulton, 2004) as adopted in SplitsTree software (Huson, 1998).

### Statistical analysis

Descriptive analysis data calculated for individual genes were used in order to compare π, θ, and ω parameters for the *p*VGC with regard to different serotypes, geographical origin or isolation source using Student's *t* test (JMP version 9.0); significance level was set at α = 0.05.

## Results

Among the 36 strain sequences analyzed, representing distinct PFGE profiles (Supplementary Material), 26 unique alleles were identified for *p*VGC (Table 3, Supplementary Dataset S1). Twenty-three isolates harbored a full length cluster of 7,503 nucleotides; 12 isolates had a 105 bp deletion in their *actA* sequence and as such had a 7,398 bp *p*VGC; one isolate had a single nucleotide deletion in its *actA* gene sequence and thus a 7,502 bp *p*VGC.

**Table 3:** Sequence diversity analysis of virulence genes sequences.

**Gene (length in nt)**	**Strains**	**Polymorphic sites**	**Substitutions**	**Alleles**	**G + C content (%)**	**SynSubs^a^**	**Non-SynSubs^b^**	**π/site^c^**	**θ/site^d^**	**Tajima's *D* value^e^**	**d_N_/d_S_**
*p*VGC (7503)	36	439	463	26	37.2	281	182	0.02427	0.01601	2.05558^**^	0.188618
Serotype 1/2a	18	243	258	14	37.2	124	134	0.00913	0.01059	−0.62147	0.230547
Serotype 4b	18	75	75	12	37.2	24	51	0.00411	0.00345	0.89916	0.169137
Food associated	23	433	456	19	37.2	276	180	0.025	0.01736	1.85522^*^	0.181073
Clinical	13	351	359	11	37.2	223	136	0.02393	0.0174	1.81904^*^	0.168402
Australian	12	405	424	10	37.2	258	166	0.02593	0.01978	1.55772	0.183604
Greek	12	373	374	8	37.2	237	137	0.02513	0.01971	1.5115	0.175085
Irish	12	384	395	11	37.2	245	150	0.0243	0.01823	1.61429	0.173244
*prfA* (711)	36	24	24	8	33.4	20	4	0.01551	0.01296	1.03014	0.0842314
Serotype 1/2a	18	6	6	5	33.4	3	3	0.00336	0.00403	−1.14554	0.26142
Serotype 4b	18	2	2	3	33.2	1	1	0.00187	0.00187	NA^f^	0.259992
Food associated	23	24	24	8	33.2	20	4	0.01551	0.01296	1.03014	0.0842313
Clinical	13	19	19	4	33.3	19	0	0.01727	0.01451	1.94585	5.00*E*−09
Australian	12	21	22	5	33.4	19	3	0.01653	0.01412	1.26346	0.0464163
Greek	12	20	20	5	33.3	19	1	0.01625	0.01345	1.54012	0.0312951
Irish	12	20	20	5	33.4	19	1	0.01597	0.01345	1.38611	0.0314107
*plcA* (951)	36	57	58	13	35.8	41	17	0.02215	0.01925	0.67574	0.166368
Serotype 1/2a	18	38	39	10	35.7	26	13	0.01624	0.01408	0.74193	0.193846
Serotype 4b	18	6	6	3	36.1	5	1	0.00419	0.00419	NA	0.0560234
Food associated	23	56	57	11	35.8	41	16	0.02314	0.02004	0.73176	0.152218
Clinical	13	50	50	7	35.8	36	14	0.02296	0.02139	0.42397	0.16682
Australian	12	51	52	8	35.8	38	14	0.02242	0.02062	0.47162	0.141993
Greek	12	43	43	5	35.7	32	11	0.02432	0.02164	0.93385	0.123106
Irish	12	54	55	8	35.9	39	16	0.02538	0.02183	0.87667	0.153798
*hly* (1587)	36	57	59	19	36	53	6	0.01409	0.01044	1.43362	0.0660299
Serotype1/2a	18	22	23	11	36	19	4	0.00453	0.00472	−0.18884	0.106618
Serotype 4b	18	31	31	8	36	26	5	0.00984	0.00752	1.63658	0.0630305
Food associated	23	55	57	16	36	51	6	0.01388	0.01061	1.30974	0.0681005
Clinical	13	51	52	9	36	48	4	0.01544	0.0118	1.57407	0.0634322
Australian	12	53	54	8	36	49	5	0.01388	0.0131	0.32216	0.0474897
Greek	12	51	51	6	36	46	5	0.01694	0.01405	1.3193	0.0498417
Irish	12	48	48	9	36	44	4	0.01328	0.01111	0.99492	0.0458643
*mpl* (1497)	36	86	87	14	38.1	58	29	0.02413	0.01873	1.16267	0.133937
Serotype1/2a	18	12	12	10	38.2	9	3	0.0026	0.00283	−0.37581	0.0731896
Serotype 4b	18	9	9	4	37.9	6	3	0.00345	0.00328	0.52223	0.118136
Food associated	23	86	87	11	38.1	58	29	0.02633	0.01984	1.56388	0.136483
Clinical	13	79	79	7	38.1	52	27	0.02418	0.02154	0.71271	0.144224
Australian	12	81	82	8	37.1	54	28	0.02221	0.02113	0.27887	0.141179
Greek	12	82	82	6	38.1	54	28	0.03059	0.02399	1.77607	0.148356
Irish	12	78	78	8	38.1	52	26	0.02667	0.0201	1.77542^*^	0.136472
*actA* (1890)	36	174	190	20	40.1	82	108	0.03782	0.029	1.26319	0.288458
Serotype1/2a	18	86	93	13	38.8	44	49	0.01819	0.01594	0.64095	0.276396
Serotype 4b	18	24	25	7	40.2	13	12	0.0055	0.00572	−0.21918	0.248332
Food associated	23	169	184	16	40.1	81	103	0.03939	0.03005	1.35214	0.280161
Clinical	13	140	152	8	40.1	61	91	0.03874	0.03549	0.50002	0.320893
Australian	12	154	167	10	40.2	72	95	0.04118	0.03228	1.37403	0.305107
Greek	12	135	142	7	40.1	61	81	0.03727	0.03156	1.06034	0.284034
Irish	12	161	175	8	40.1	78	97	0.04197	0.03614	0.88101	0.288721
*plcB* (870)	36	45	46	12	36	26	20	0.02254	0.01751	1.31427	0.189206
Serotype1/2a	18	8	8	8	35.8	4	4	0.00271	0.00355	−1.14142	0.246995
Serotype 4b	18	4	4	4	36.3	2	2	0.0023	0.00251	−0.78012	0.251206
Food associated	23	45	46	11	35.9	26	20	0.02082	0.01805	0.72105	0.188388
Clinical	13	41	42	6	36	25	17	0.02713	0.02114	1.80741^*^	0.16398
Australian	12	42	43	8	35.9	24	19	0.02011	0.01906	0.29607	0.189685
Greek	12	43	44	6	36.1	26	18	0.02797	0.02215	1.67957	0.168418
Irish	12	40	41	6	36.1	24	17	0.02674	0.02064	1.88777	0.169274

a *Number of synonymous substitutions*.

b *Number of non-synonymous substitutions*.

c *Average pairwise nucleotide difference per site*.

d *Index of the number of segregating sites (mutation rate)*.

e Tajima's D-values significantly different from 0 are indicated with

* (0.05 < P < 0.1) or with

** *(P < 0.05)*.

f *The value is not available, as it could be not evaluated due to the low number of alleles (4 or more sequences needed)*.

**Figure 1:**
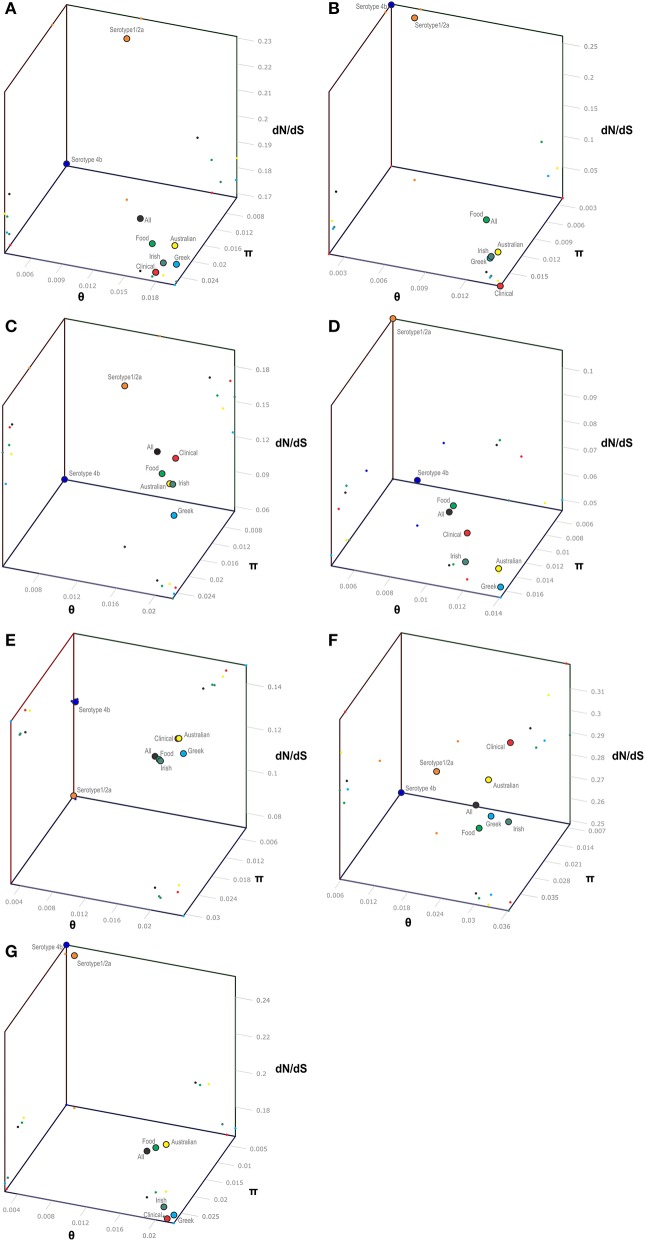
3-D scatter-plot illustration of nucleotide diversity parameters (π, θ) and d_N_/d_S_ ratio (ω) for the *p*VGC **(A)**, *prfA* **(B)**, *plcA* **(C)**, *hly* **(D)**, *mpl* **(E)**, *actA* **(F)**, and *plcB* **(G)** genes. Within each gene, colored dots represent the *L. monocytogenes* population grouping based on serotype (4b and 1/2a), geographical origin (Australian, Greek, and Irish strains), source of isolation (clinical or food environment), and as a whole (All strains).

**Figure 2:**
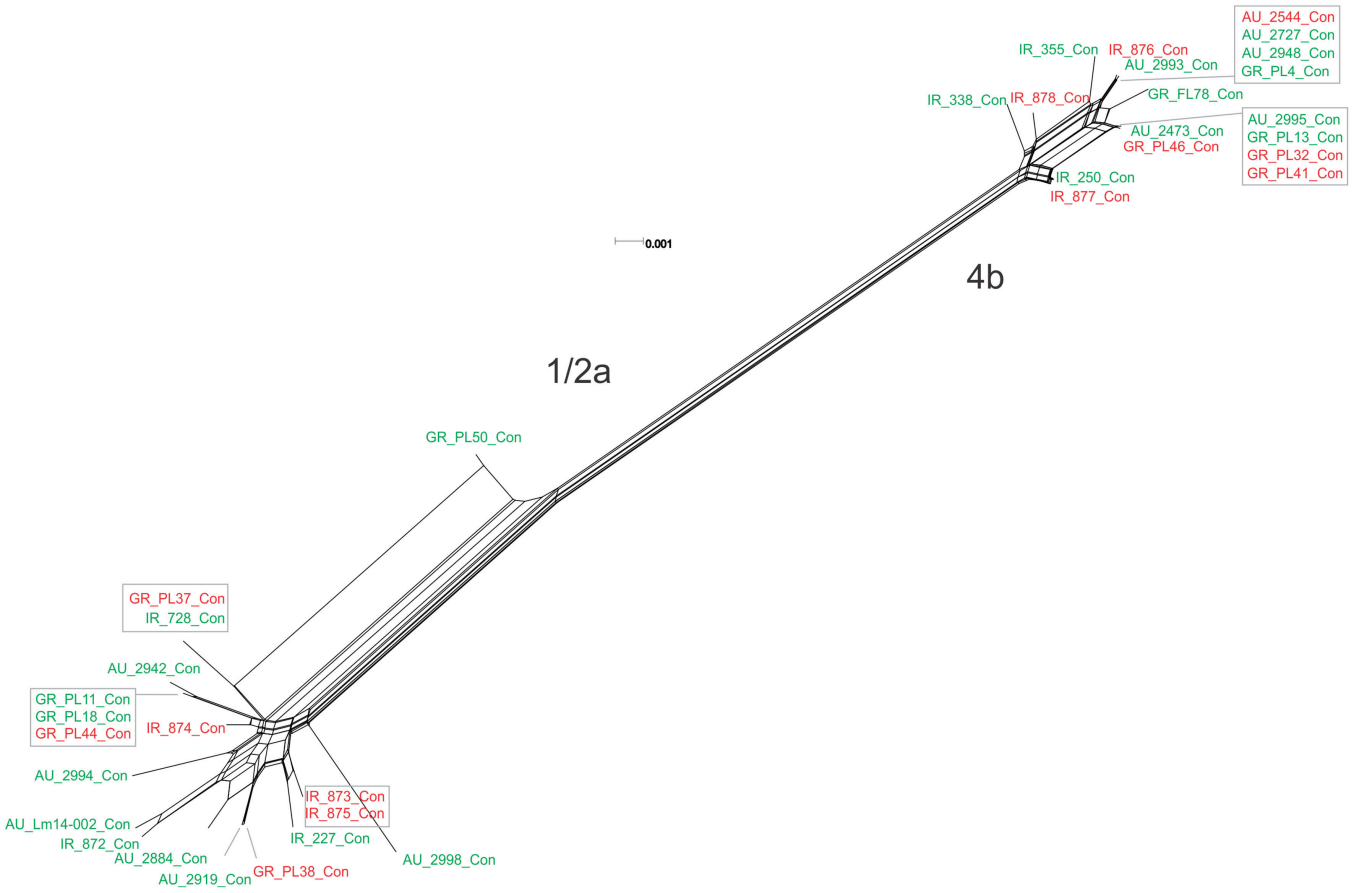
Phylogenetic network applied to virulence gene cluster *prfA* (*p*VGC; concatenated genes *prfA, plcA, hly, mpl, actA*, and *plcB*) using the Neighbor-Net algorithm. *L. monocytogenes* strains represented food environment isolates (green color) or clinical isolates (red color), isolated in Ireland (IR), Greece (GR), and Australia (AU). Strains clustered together in a box represent identical nucleotide sequence.

The *p*VGC contained 439 polymorphic sites, with 281 synonymous and 182 non-synonymous substitutions. The G + C% content was 37.2%. The overall nucleotide diversity was π = 0.02427 and θ = 0.01601. Although π and θ values for serotype 1/2a strains were higher than for 4b strains, the difference was not significant (*P* > 0.05). No significant π difference was observed among strains of different geographical origin, or between food environment and clinical origin. Comparing groupings by serotype, geographical origin or isolation source, grouping by serotype had a significantly lower π value (*P* < 0.0001). Serotype groups also exhibited distinct clustering on the 3D-scatter plot (Figure 1A), showing divergence from the other groupings. Divergence between the two serotypes in d_N_/d_S_ ratio was also observed, suggesting different selective pressure acting on the two serotypes, with higher values among the serotype 1/2a group. The *p*VGC phylogenetic tree (Figure 2) showed two major distinct clusters representing the two serotypes, 1/2a and 4b. No specific pattern of origin-based classification was observed, with strains isolated in different countries or from different sources (i.e., food-associated or clinical) sharing an identical nucleotide sequence. In each serotype group, stains were clustered in short distances to each other, with only strain GR_PL50 distant to the others.

**Figure 3:**
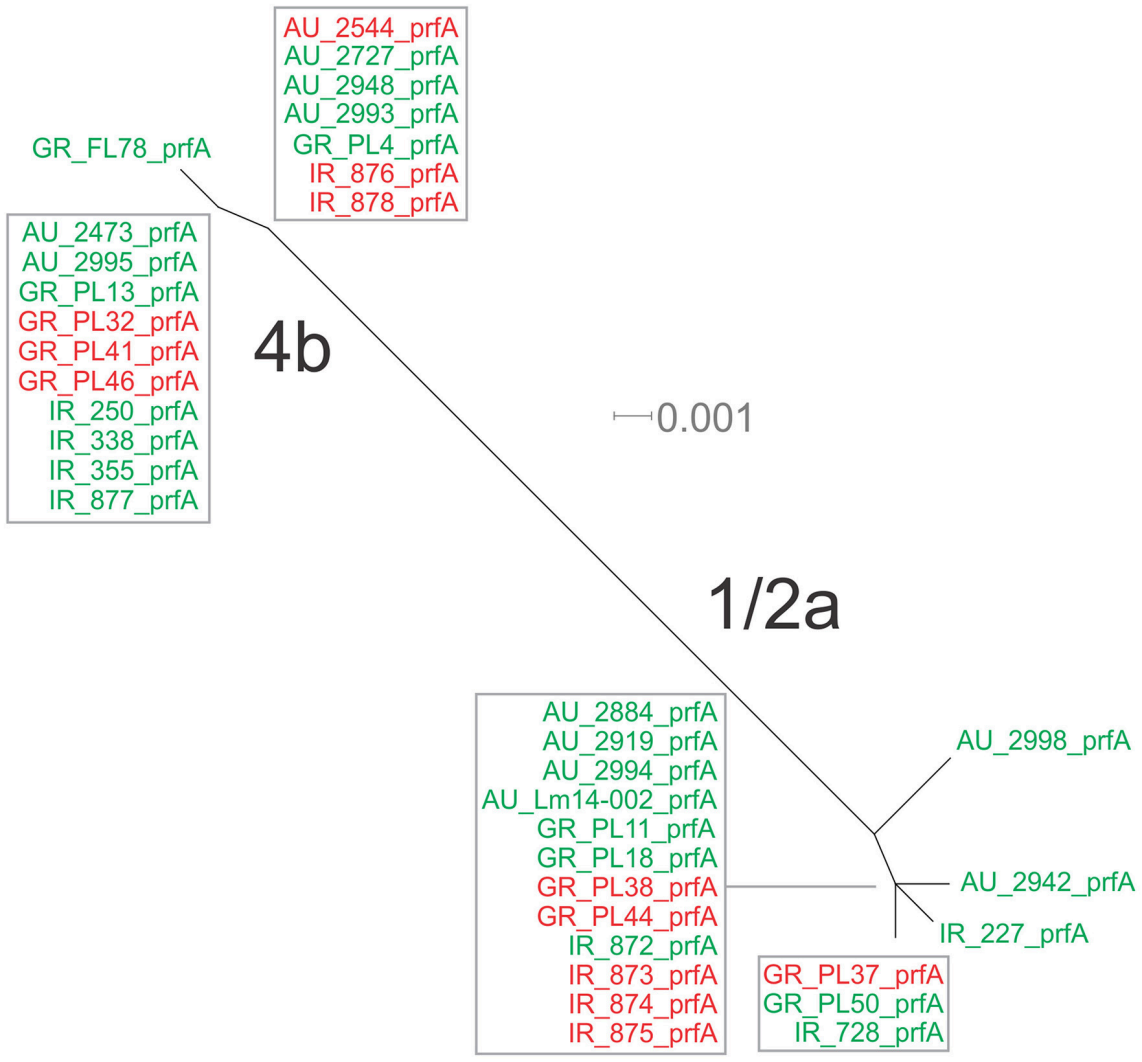
Phylogenetic network applied to virulence gene *prfA* using the Neighbor-Net algorithm. *L. monocytogenes* strains represented food environment isolates (green color) or clinical isolates (red color), isolated in Ireland (IR), Greece (GR), and Australia (AU). Strains clustered together in a box represent identical nucleotide sequence.

Eight haplotypes among the 36 strains were recovered for the *prfA* gene (5 for 1/2a serotype and 3 for 4b serotype). This gene possessed the lowest number of polymorphic sites (*n* = 24) with the lowest number of substitutions (*n* = 24) and non-synonymous substitutions (*n* = 4) compared to all fragments tested (Table 3). The overall nucleotide diversity was π = 0.01551 and θ = 0.01296. Groups containing strains of different geographical origin were clustered closely to each other (Figure 1B), while food isolates were distinct from the clinical isolates with respect to π values. Divergence in d_N_/d_S_, π and θ parameters resulted in distinct clustering of serotype groups compared to other groupings. The phylogenetic tree of *prfA* gene (Figure 3) showed the lowest degree of divergence among all tested genes, with longer branch lengths observed for 1/2a serotype isolates than for 4b isolates, which is in accordance with the higher nucleotide diversity within 1/2a serotype than 4b serotype (Table 3). Among the 19 substitutions observed for clinical isolates, none of them were non-synonymous.

**Figure 4:**
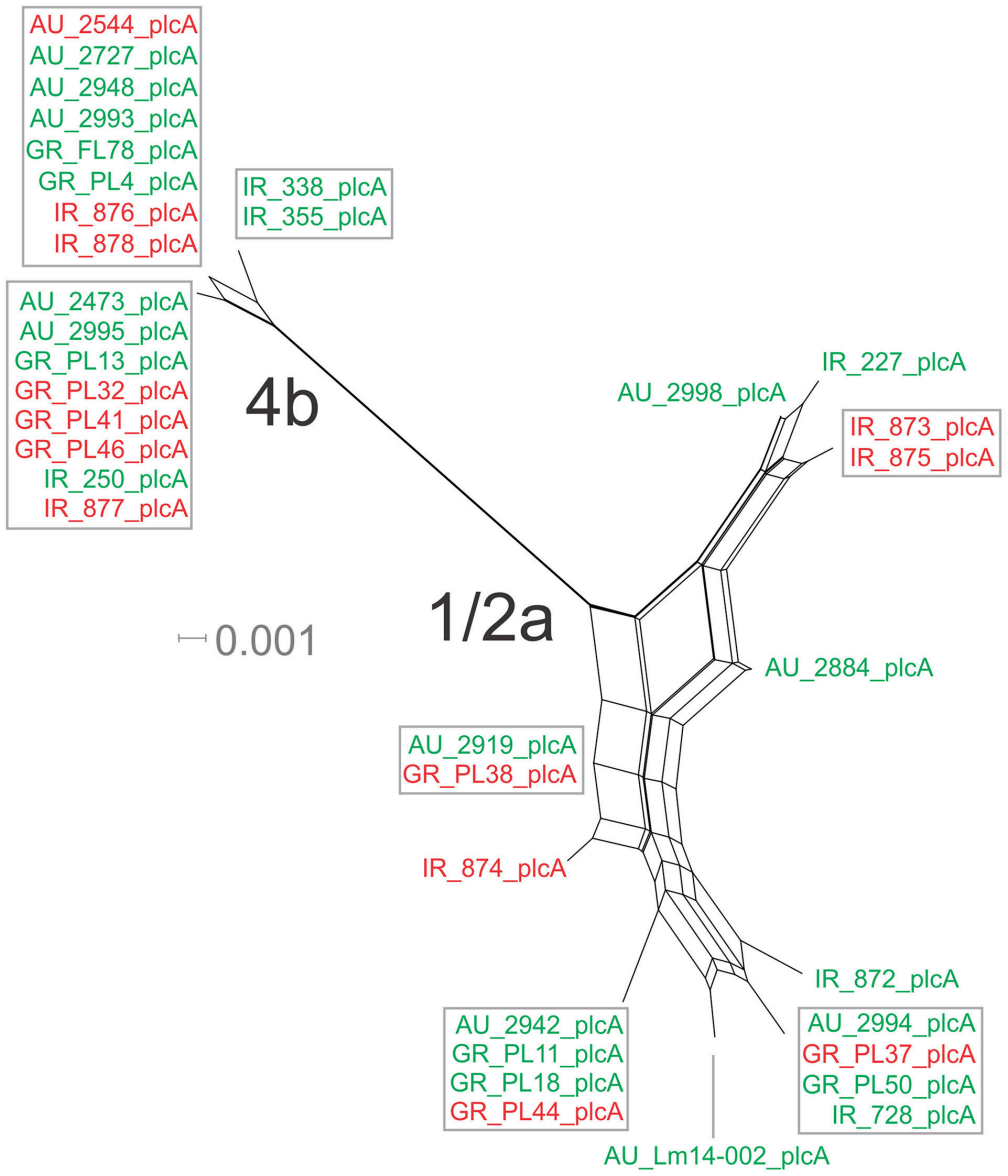
Phylogenetic network applied to virulence gene *plcA* using the Neighbor-Net algorithm. *L. monocytogenes* strains represented food environment isolates (green color) or clinical isolates (red color), isolated in Ireland (IR), Greece (GR), and Australia (AU). Strains clustered together in a box represent identical nucleotide sequence.

The nucleotide sequence of the *plcA* gene (13 haplotypes; π = 0.02215) was diversified into 10 unique alleles of 1/2a serotype strains (π = 0.01624) and 3 alleles of 4b serotype strains (π = 0.0419). Serotype 4b strains had the lowest number of substitutions (*n* = 6) compared to the other subgroups (*n* = 39–57), which resulted in the lowest nucleotide diversity. Serotype 1/2a strains differed from the other groups in d_N_/d_S_ ratio values and serotype 4b strains in θ values, resulting in distinct clustering on the 3D-scatter plot (Figure 1C). The phylogenetic tree of the *plcA* gene (Figure 4) showed that isolates of the 1/2a serotype were highly divergent with more distant branches compared to 4b serotype strains. Unique sequence types in the group of 1/2a serotype belonged to Australian or Irish origin strains.

**Figure 5:**
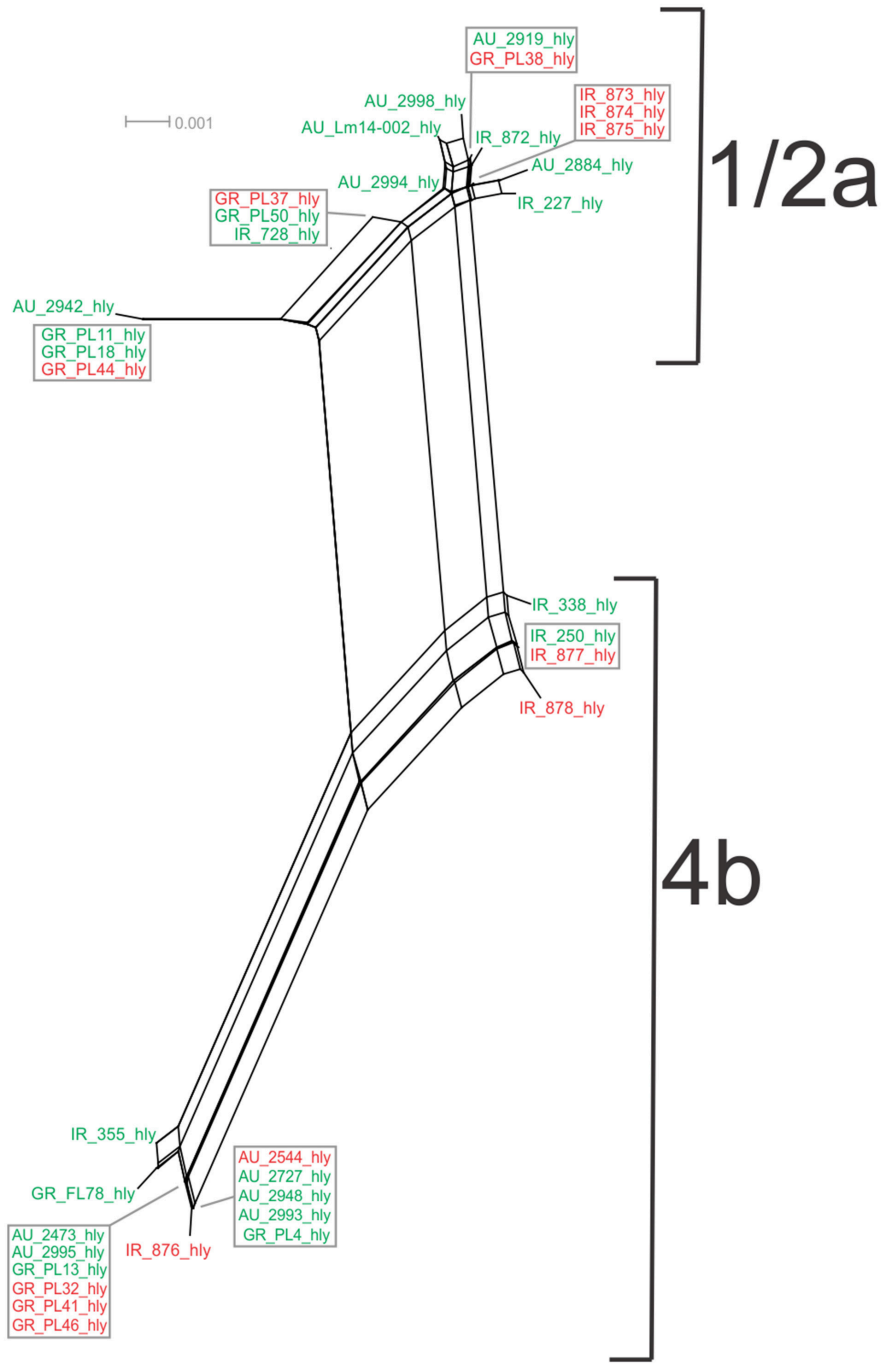
Phylogenetic network applied to virulence gene *hly* using the Neighbor-Net algorithm. *L. monocytogenes* strains represented food environment isolates (green color) or clinical isolates (red color), isolated in Ireland (IR), Greece (GR), and Australia (AU). Strains clustered together in a box represent identical nucleotide sequence.

Analysis of the *hly gene* showed 19 haplotypes among the 36 strains with overall nucleotide diversity π = 0.01409 and θ = 0.01044. Higher diversity was observed among 1/2a serotype than 4b serotype strains (11 and 8 unique alleles, respectively). Groups of different geographical origin or groups of different isolation source (i.e., food environment or clinical) were clustered closely to each other (Figure 1D), in contrast to different serotypes, where the two groups (i.e., 1/2a and 4b serotypes) clustered apart along the d_N_/d_S_ ratio axis showing a diverse selective pressure acting on the gene within each serotype. As illustrated in Figure 5, a high divergence in *hly* gene sequences among strains of 4b serotype was observed; two subpopulations were identified, one of which only included Irish isolates. The second subpopulation contained two sets of strains with shared sequences between Australian and Greek strains, and three unique alleles (i.e., one Greek strain and two Irish).

**Figure 6:**
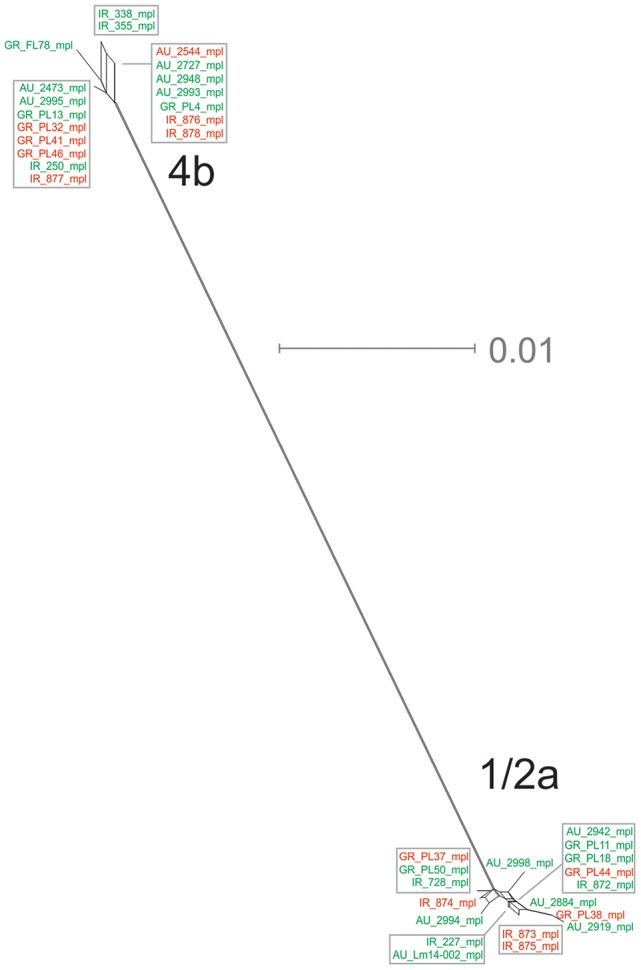
Phylogenetic network applied to virulence gene *mpl* using the Neighbor-Net algorithm. *L. monocytogenes* strains represented food environment isolates (green color) or clinical isolates (red color), isolated in Ireland (IR), Greece (GR), and Australia (AU). Strains clustered together in a box represent identical nucleotide sequence.

The *mpl* gene was represented by 14 unique alleles, 10 for 1/2a serotype and 4 for 4b serotype, with π = 0.02413 and θ = 0.01873. Grouping according to serotypes resulted in distinct clusters compared to the other groupings (Figure 1E), due to lower π values, while additionally the two serotype groups (i.e., 1/2a and 4b) differed in their d_N_/d_S_ ratio demonstrating diverse selective pressure acting on the strains of each serotype within this gene. The phylogenetic tree for the *mpl* gene (Figure 6) showed a similar clustering of the strains between the two serotypes with respect to branch lengths, and higher divergence within the 1/2a serotype compared to 4b serotype, in terms of unique alleles.

**Figure 7:**
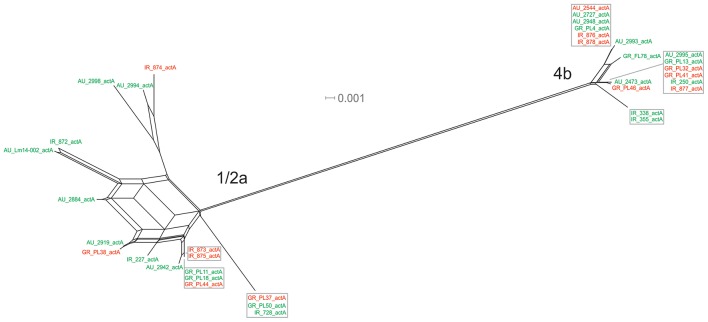
Phylogenetic network applied to virulence gene *actA* using the Neighbor-Net algorithm. *L. monocytogenes* strains represented food environment isolates (green color) or clinical isolates (red color), isolated in Ireland (IR), Greece (GR), and Australia (AU). Strains clustered together in a box represent identical nucleotide sequence.

The *actA* gene was represented by 20 unique alleles, the highest number among any of the *p*VGC genes, with overall nucleotide diversity π = 0.03782 and θ = 0.029. Groups containing strains of various origins or serotypes were highly variant, as illustrated in Figure 1F, confirming the diversity of this particular gene. Strains of serotype 1/2a were more diverse (π = 0.01819, θ = 0.01594) than serotype 4b strains (π = 0.0055, θ = 0.00572). This was also evident from the phylogenetic tree (Figure 7), where 13 different nucleotide sequences were found among 18 isolates of 1/2a serotype, with longer branch lengths compared 4b serotype strains. Food isolates had the highest number of non-synonymous substitutions (*n* = 103) among all subgroups within this gene and clinical isolates the lowest (*n* = 12). A large variation between the d_N_/d_S_ ratio values was observed for food and clinical isolates, suggesting a different selective pressure acting on these two groups. Divergence in d_N_/d_S_ was also observed between Australian and Greek or Irish isolates. Twelve isolates, representing 5 unique alleles, had a 105-bp deletion in their sequences; 8 of these isolates were of food environment origin and 4 of clinical origin. The isolate (AU_Lm14-002) that had a single nucleotide deletion was of food origin.

**Figure 8:**
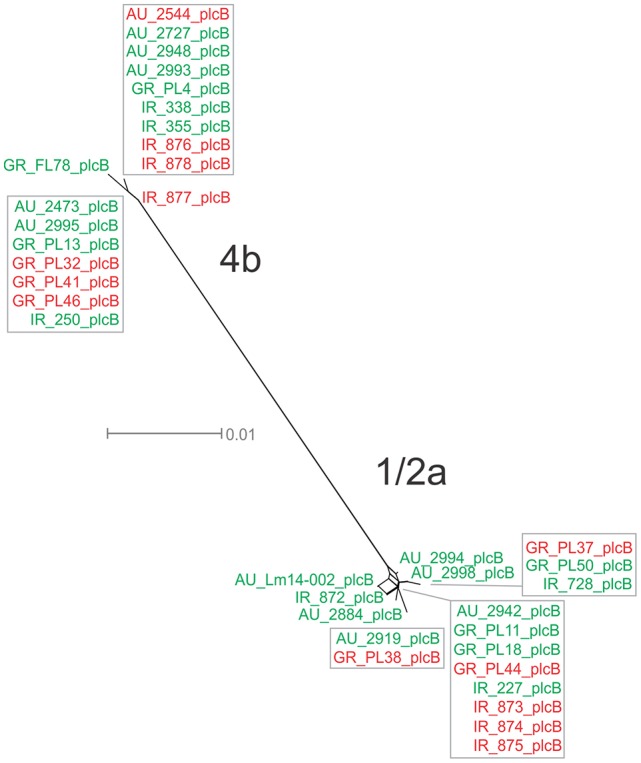
Phylogenetic network applied to virulence gene *plcB* using the Neighbor-Net algorithm. *L. monocytogenes* strains represented food environment isolates (green color) or clinical isolates (red color), isolated in Ireland (IR), Greece (GR), and Australia (AU). Strains clustered together in a box represent identical nucleotide sequence.

For the *plcB* gene, 12 haplotypes were observed among the 36 strains, with nucleotide diversity π = 0.02254 and θ = 0.01751. Serotype 1/2a strains were more diverse than 4b strains, represented by higher numbers of unique alleles (8 and 4, respectively), and higher π and θ values. Food-related strains differed from clinical strains, and Australian strains clustered apart from Greek and Irish strains (Figure 1G), showing lower nucleotide diversity and thus, a higher genetic uniformity within the former groups (i.e., food or Australian) compared to the latter (i.e., clinical, Greek, or Irish). In the phylogenetic tree (Figure 8), the short length of the branches indicated the small divergence level among strains within each serotype.

Comparing all genes, the most diverse gene was *actA* (π = 0.03782) and the most conserved *hly* (π = 0.01409) and *prfA* (π = 0.01551); the π value of *actA* was significantly higher compared to *hly* (*P* = 0.0095) or *prfA* (*P* = 0.0088). Additionally, for *p*VGC no significant difference in nucleotide diversity was observed between the two serotype groupings, the two isolation sources or the three geographical origin groups. Higher nucleotide diversity in serotype 4b vs. serotype 1/2a was only observed for *mpl* and *hly* genes. Regarding the selective pressure acting on the genes, the highest values of the d_N_/d_S_ ratio were observed for *actA* and the lowest on *prfA* and *hly* genes (*P* < 0.05).

Tajima's *D*-test for neutrality (Tajima, 1989; Simonsen et al., 1995), which examines whether the occurring mutations are a result of selection or random (neutral) evolution, showed a significantly positive value for the test for the *p*VGC (Table 3). This suggests that the gene evolution deviates significantly from the standard neutral model and is under balancing selection, decrease in population size or a subdivision of the population structure. High Tajima's *D*-values (0.1 > *P* > 0.05) were also observed for food and clinical isolates in the *p*VGC, for Irish isolates in the *mpl* gene and for clinical isolates in the *plcB* gene. Negative values were observed for serotype 1/2a strains in the *p*VGC, *prfA*, and *plcB* genes, and for 4b serotype in *plcB*; however, these were not statistically significant and therefore are unlikely to represent a population bottleneck, a selective sweep or purifying selection.

## Discussion

In the present study, the intraspecies variations in the *prfA* virulence gene cluster among 36 *L. monocytogenes* strains, with respect to different serotype (i.e., 1/2a and 4b), geographical origin (Australian, Greek, and Irish isolates), or isolation source (i.e., food environment or clinical isolates) was investigated. Consistent with previous classification studies (Ward et al., 2004; Orsi et al., 2008), within all six virulence genes analyzed and the *p*VGC, strains were divided into two major clusters, each representing one serotype, i.e., 4b and 1/2a serotype, which belong to lineage I and II, respectively. *L. monocytogenes* is a highly diverse species and lineages I and II are considered to be deeply separated evolutionary lineages (Nightingale et al., 2005). Significant association between lineage and the origin of the strains has been reported (Wiedmann et al., 1997), while additionally, molecular types of the strains were shown to be associated with specific food types (Gray et al., 2004). Strains of different lineages are also divergent in terms of their virulence potential. While higher virulence associated with the lineage I population relative to that of lineage II has been reported, (Wiedmann et al., 1997; Norton et al., 2001; Gray et al., 2004; Jensen et al., 2007), others found no statistical correlation between virulence of the strains and their serotypes (Conter et al., 2009). Therefore, molecular typing and a better understanding of virulence stratification among serotypes and lineages are essential in epidemiological surveys and risk estimation procedures. The analysis in this study also showed that 4b serotype strains exhibited lower diversity than the 1/2a strains. This is consistent with previous findings where lineage II strains were genetically more diverse compared to lineage I, based on molecular typing of seven genetic loci including four housekeeping genes, two virulence genes and stress response *sigB* gene (den Bakker et al., 2008), ribotyping and random multiprimer PCR analysis (Mereghetti et al., 2002), or analysis of the *prfA* virulence gene cluster (Orsi et al., 2008). In addition to these reports, it was shown here that ω values were similar between the serotype groups for *prfA* and *plcB*, while varied largely for the *p*VGC*, plcA, hly, mpl*, and *actA*, indicating a different selective pressure acting on these genes within each serotype. Furthermore, the opposite (i.e., negative vs. positive) Tajima's *D* values for the serotype groups within *p*VGC, *hly, mpl*, and *actA* suggest that these genes follow a different evolutionary pathway across serotypes.

Results of this study showed that among the six genes examined, only the *hly* gene of 4b serotype strains was partially correlated with geographical origin, with strains separating into two distinct subpopulations: one containing only Irish strains, the other containing Greek and Australian strains and two Irish strains. Since serotype 4b strains have been found as the etiological agent of the majority of epidemic or sporadic human listeriosis cases in many countries, including Ireland (Schuchat et al., 1991; Swaminathan and Gerner-Smidt, 2007; Fox et al., 2012), and *hly* is a key gene for the virulence potential of *L. monocytogenes* (Gaillard et al., 1986; Roberts et al., 2005), the correlation between Irish strains and *hly* could imply a possible impact of geographical-specific diversification. Previous studies have shown no polymorphism in LLO protein among 150 strains of food and human origin, while slight changes in the *hly* gene did not imply alterations on LLO molecular weight (Jacquet et al., 2002). Furthermore, no significant differences in the LLO protein among different serotypes 4b and 1/2a were reported (Matar et al., 1992; Jacquet et al., 2002). Nonetheless, Gray et al. (2004) reported a significant correlation between *hly* allelic types and origin of the strains (i.e., food vs. human isolates); *hly* type 1 was significantly more common among human isolates and was associated with larger plaque forming, indicative of *in vitro* cytopathogenicity, compared with other *hly* types (Gray et al., 2004). Therefore, such correlation of origin and *hly* types might be important in epidemiological studies. Additionally, studies based on ribotype analysis showed no specific clustering among *L. monocytogenes* strains distributed across different geographical locations, and therefore no significant effect of geographical distribution on their genetic diversity (Gendel and Ulaszek, 2000; Jaradat et al., 2002; Mereghetti et al., 2002). The sequence diversity analysis in the current study showed that the groups of Greek, Australian and Irish isolates within the *p*VGC form distinct clusters based on parameters π, θ, and ω, which may underlie diverse evolutionary pathways for each group; this was also observed for all individual genes except the *prfA* gene. Origin-based pattern in nucleotide diversity was observed for Australian strains, which showed less diversity in *plcB* and *mpl* sequences relative to their Irish or Greek counterparts. The Tajima's *D*-values for Australian isolates were close to 0 contrary to Greek and Irish isolates with increased Tajima's *D*-values. This indicates a differentiation in the evolutionary pathway of Australian compared to Greek and Irish isolates within these genes.

Although serotype 4b strains predominate among human clinical isolates and serotype 1/2a strains among food isolates, gene-specific pattern between clinical isolates and 4b serotype strains or between food isolates and 1/2a serotype strains were not observed; food and clinical isolates could share alleles for all genes tested. However, descriptive analysis revealed that food and clinical isolates formed distinct clusters regarding their π and ω parameters for all the genes tested, with larger variations within *prfA, actA*, and *plcB* genes. This divergence might indicate that these genes were adapted differentially within each group, and this adaptation correlated with their prevalence in food or virulence phenotype, respectively. Previous studies investigating the correlation of isolation source and virulence of strains yielded differing conclusions. Some showed lower virulence potential for strains isolated from food environments compared to human clinical isolates (Norton et al., 2001; Jensen et al., 2008). Conversely, Larsen et al. (2002) reported no significant correlation between food or human origin of strains and invasiveness in the Caco-2 cell infection model, while all strains managed equally to multiply once inside the host cells when an *in vivo* test was used. Similarly others found no systematic differences in virulence between food or clinical isolates (Brosch et al., 1993; Gray et al., 2004; Neves et al., 2008; Bueno et al., 2010).

The results of this study also showed that the most conserved genes were *prfA* and *hly* and the most diverse was *actA*. Proteins PrfA, LLO and ActA are considered essential virulence factors (Gaillard et al., 1986; Nishibori et al., 1995; Vázquez-Boland et al., 2001a; Travier and Lecuit, 2014). It seems that there is a selective pressure on *L. monocytogenes* to maintain the former genes, while the increased diversity of *actA* compared to the other genes is consistent with previous findings (Orsi et al., 2008) and is attributed to increased recombination events occurring in *actA*, and to evolution by positive selection in both lineages I and II. Rapid PCR-based methods utilize species-specific genes to detect *L. monocytogenes* in food samples, aiming at preventing the unnecessary recalls of food products. It is of great importance to use target sequences of highly conserved regions rather than genes prone to genetic variability (Rodríguez-Lázaro et al., 2004). Virulence associated genes (e.g. *actA, hly, inlA, inlB, prfA, plcA, plcB*) and 16S/23S rRNA genes have been studied toward the development of such methods (Liu, 2006). The results indicated that due to the diversity seen, PCR assays based on *prfA* or *hly* as opposed to *actA* would be more reliable, covering isolates of different origin, serotype or isolation source.

In the current study, *actA* showed the highest number of alleles among all genes tested; 13 alleles were observed for serotype 1/2a strains and 7 alleles for serotype 4b strains. One food isolate (AU_Lm14-002) had a single nucleotide deletion. Although this deletion would lead to a premature stop codon and a predicted truncated 487 amino acid protein, it was located immediately upstream of a poly(A) tract of 7 adenine residues. These mutations may have a role in influencing gene regulation, which allows phase switching and inactivation, and may be influencing *actA* transcription in this isolate, whereby a full length ActA may still be synthesized (Orsi et al., 2010). Twelve isolates representing 5 unique alleles had a 105 bp deletion, which comprises a 35 amino acid Proline-Rich Repeats (PRRs) fragment (Wiedmann et al., 1997; Jacquet et al., 2002; Orsi et al., 2008; Holen et al., 2010); the encoded proteins possess 3 instead of 4 PRRs. The number of PRRs contributes to bacterial movement (Lasa et al., 1995; Smith et al., 1996), however no significant effect on virulence potential of the strains has been shown (Roberts and Wiedmann, 2006; Holen et al., 2010). Among the isolates tested in this study, the 105 bp deletion was observed for 4 out of 18 isolates of 1/2a serotype and 8 out of 18 isolates of 4b serotype. Of these, 8 strains (which includes 3 alleles) were isolated from the food environment and 4 strains (2 alleles) were clinical isolates. Similar results were demonstrated by Wiedmann et al. (1997), who observed a predominance of 3-PRRs *actA* sequence among lineage I isolates compared to isolates of lineage II. This could indicate that this deletion does not influence the pathogenic potential of *L. monocytogenes*. Jacquet et al. (2002) observed that polymorphism in ActA proteins was rather correlated with origin (human or food isolates) than with serotype of the strains, while Conter et al. (2009) could not correlate *actA* polymorphism to the virulence of the strains. Based on the sequence analysis in the current study, no clear driving factor appeared to influence the nucleotide sequence or mutations in this gene, as all of the groups were dispersed regarding the parameters π, θ, and ω, while phylogenetic trees showed no consistent pattern between origin or environment of the strains and their genetic polymorphisms. These findings, along with the adapting character to certain functions previously suggested for this gene, and the increased recombination events (Orsi et al., 2008) might imply its multi-functionality recently reported (Travier et al., 2013).

Overall, this study provides insights into the selective pressures acting on the main virulence gene cluster of *L. monocytogenes*, and suggests differences based on serotype, geographic location and source. The selective pressure to minimize diversification was noted with the key virulence regulatory gene *prfA*, therefore results of this study support the key role of the global regulator *prfA* in the lifecycle of *L. monocytogenes*. In contrast to this, conservation of the *actA* gene sequence was lowest, with a greater sequence variation and number of alleles. Broadly speaking, higher conservation was noted among isolates sharing a serotype when compared with other groupings such as geographical location or source. Food and clinical isolates largely varied with respect to nucleotide diversity within *prfA, actA*, and *plcB* genes, possibly suggesting that a particular adaptation correlated with their prevalence in food or virulence phenotype, respectively. Geographical divergence was noted with respect to the *hly* gene, with serotype 4b Irish strains distinct to Greek and Australian strains. Future studies will be needed in order to clarify the correlation of geographical distribution of strains and their *hly* sequence, as well as the impact of such correlation on LLO functionality. Additionally, *actA* polymorphism should be further evaluated for other phenotypes that might result from its increased diversity among strains and diverse origins. In the present study, strains were selected to represent the distribution of *L. monocytogenes* based on prevalent serotypes and clinical or food associated origin. Further, a larger data set comprising strains of more serotypes, geographical or isolation origin and year of isolation should be investigated in order to infer significant conclusions regarding the impact of these parameters on LIPI-1 evolution and its correlation to virulence potential of the pathogen.

## Author contributions

The study was conceived and designed by KJ, PS, and EF. All authors contributed to acquisition, analysis, and interpretation of the data. The work was drafted and revised by SP and EF. All authors approved and agreed in the final version of the manuscript.

### Conflict of interest statement

The authors declare that the research was conducted in the absence of any commercial or financial relationships that could be construed as a potential conflict of interest.
