# Supplementary material for: Virulence Gene Sequencing Highlights Similarities and Differences in Sequences in Listeria monocytogenes Serotype 1/2a and 4b Strains of Clinical and Food Origin From 3 Different Geographic Locations
Source: Front Microbiol. 2018 Jun 5;9:1103. doi: 10.3389/fmicb.2018.01103 (PMC5996115; doi:10.3389/fmicb.2018.01103)

Supplementary Material

Virulence gene sequencing highlights similarities and differences in sequences in serotype 1/2a and 4b strains of clinical and food origin from 3 different geographic locations

Sofia V. Poimenidou[1], Marion Dalmasso[2]*, Konstantinos Papadimitriou[3], Edward M. Fox[4], Panagiotis N. Skandamis[1] and Kieran Jordan[2]**

[1] Laboratory of Food Quality Control and Hygiene, Department of Food Science and Human Nutrition, Agricultural University of Athens, Athens, Greece

[2] Teagasc Food Research Centre, Moorepark, Fermoy, Co. Cork, Ireland

[3] Laboratory of Dairy Research, Department of Food Science and Human Nutrition, Agricultural University of Athens, Athens, Greece

[4] CSIRO Animal Food and Health Sciences, Werribee, VIC, Australia

* Current address: Normandie Univ, UNICAEN, ABTE, 14000 Caen, France

**** Correspondence:**

Kieran Jordan: Kieran.Jordan@teagasc.ie

Supplementary Figure 1. Dendrogram of the PFGE profiles from isolates used in the study. Thirty-six *L. monocytogenes* strains were analysed representing three distinct geographically dispersed regions (Australia, AU; Greece, GR; Ireland, IR), two serotypes (serotype 4b and 1/2a) and two isolation sources (clinical and food–related isolates).

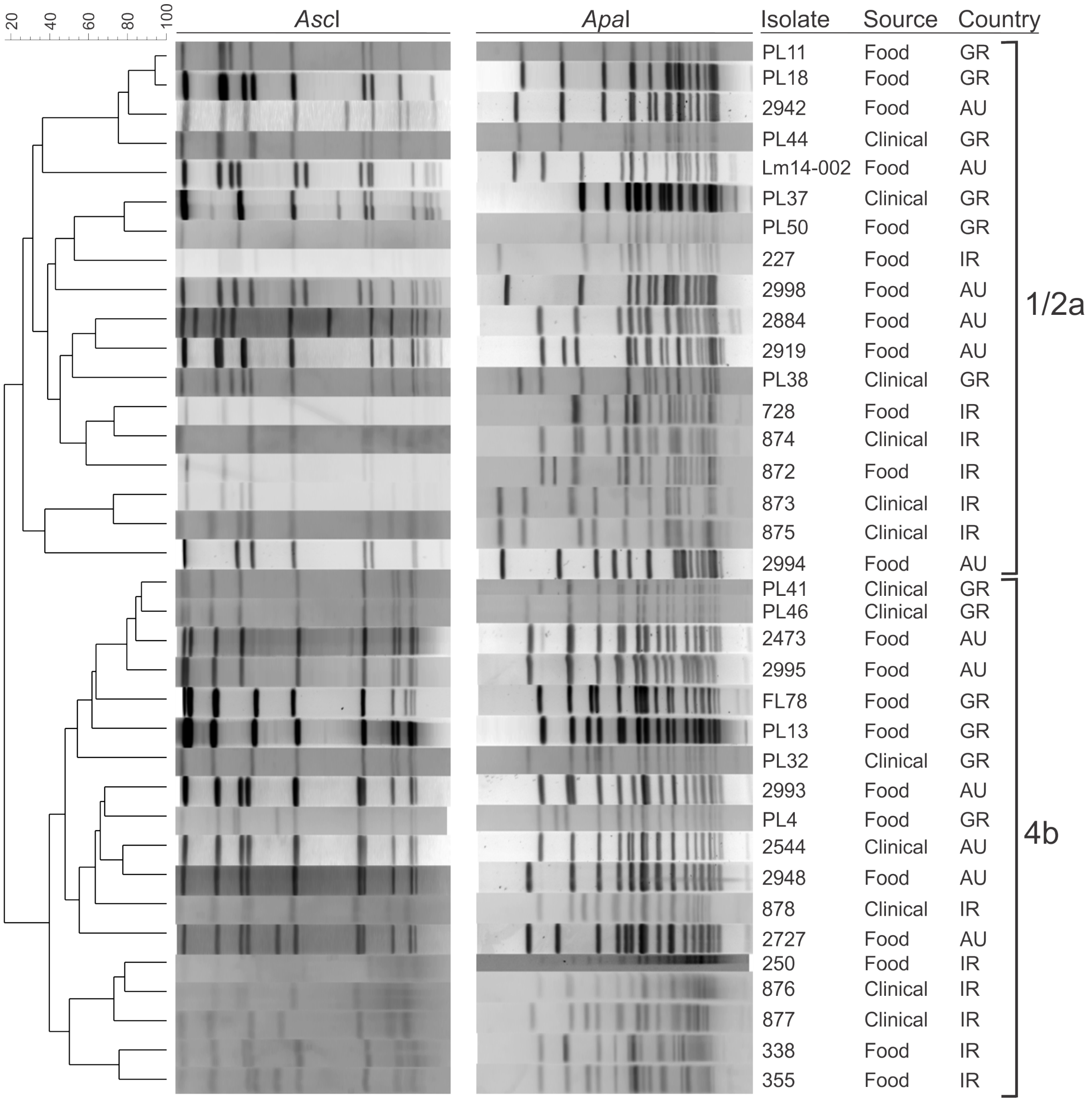

Supplement: Supplementary file 1 [file Image_1.PDF]
